# Evaluation of serum cathepsin B, D, and L concentrations in women with late-onset preeclampsia

**DOI:** 10.4274/tjod.galenos.2019.40460

**Published:** 2019-07-03

**Authors:** Gökçe Anık İlhan, Begüm Yıldızhan

**Affiliations:** 1 Marmara University Faculty of Medicine, Department of Obstetrics and Gynecology, İstanbul, Turkey

**Keywords:** Cathepsin B, cathepsin D, cathepsin L, preeclampsia

## Abstract

**Objective:**

The aim of the study was to assess serum cathepsin B, D, and L concentrations in women with late-onset preeclampsia.

**Materials and Methods:**

One hundred forty pregnant women were enrolled in the study, of which 100 subjects were preeclamptic and 40 were healthy controls. Serum concentrations of cathepsin B, D, and L were measured and compared between the preeclamptic and control groups.

**Results:**

Cathepsin B and D concentrations were significantly higher in the preeclamptic group compared with the control group. There was no statistically significant difference between the groups in terms of cathepsin L concentrations. Cathepsin B concentrations were significantly higher in women with preeclampsia with severe features compared with those with preeclampsia alone.

**Conclusion:**

Women with late-onset preeclampsia have significantly higher serum cathepsin B and D concentrations than controls. Cathepsin B and D may be promising biomarkers in women with late-onset preeclampsia.

**PRECIS:** Cathepsin B and D may be promising biomarkers in women with late-onset preeclampsia.

## Introduction

Cathepsin proteases have been suggested to be involved in a variety of cellular processes such as apoptosis, angiogenesis, cell proliferation, and invasion^(1)^. The important roles of cathepsins have been implicated in normal and abnormal placentation;^(1)^ however, research on the serum concentrations of cathepsins in preeclampsia is limited.

Preeclampsia is one of the leading causes of maternal-fetal morbidity and mortality, affecting approximately 3-5% of all pregnancies^(2)^.Determining late-onset preeclampsia, which is more common than the early-onset preeclampsia,^(2)^ and identification of high risk individuals may be helpful for close monitoring and to minimize adverse outcomes in clinical practice. The aim of the study was to assess serum cathepsin B, D, and L concentrations in women with late-onset preeclampsia and to determine the impact of cathepsins with regard to the presence of severe features.

Cathepsin B and L are cysteine proteases that have important roles in placental development and in the physiology of normal and pathologic conditions. Cathepsin B is reported to be predominantly located in placental and decidual macrophages, which may be important in mediating villous angiogenesis and decidual apoptosis, and cathepsin L is found to be expressed in invasive cytotrophoblasts^(1)^.Both cysteine proteases are determined to have critical roles during normal placentation and in the etiology of preeclampsia^(1)^. Cathepsin D is an aspartic protease that participates in the trophoblast invasion process,^(3)^ and the contribution of cathepsin D is also suggested in the pathogenesis of preeclampsia^(4,5)^.

However, there are a limited number of studies that evaluated serum concentrations of cathepsins in preeclamptic women and the studies had small sample sizes. Additionally, to our knowledge this is the first study to evaluate serum cathepsin B, D, and L concentrations in late-onset preeclampsia.

## Materials and Methods

After obtaining written informed consent from all participants, one hundred forty pregnant women were enrolled in the study, of which 100 subjects were preeclamptic (late-onset preeclampsia diagnosed at ≥34 weeks gestation) and 40 were healthy controls. The study protocol was approved by the Marmara University Ethics Committee (approval number: 09.2017/411).

Preeclampsia and severe features were defined according to current recommendations based on the 2013 American College of Obstetricians and Gynecologists’ consensus guidelines^(6)^. The common inclusion criteria for both groups were: singleton pregnancy at ≥34 weeks gestation, normal fetal morphology, non-smoking, and the absence of concomitant disease.

Serum concentrations of cathepsin B, D, and L were measured using an enzyme-linked immunosorbent assay (ELISA) by using the human cathepsin B, D, (Elabscience, Houston, TX) and L (Invitrogen, Carlsbad, CA) ELISA kits according to the manufacturer’s instructions and then compared between the preeclamptic and control groups.

### Statistical Analysis

All data were analyzed using the Statistical Package for the Social Sciences 20.0 for Windows program (IBM SPSS Statistics for Windows, Version 20.0. Armonk, NY: IBM Corp). The distribution of data was measured using the Kolmogorov-Smirnov test. Data are presented as mean ± standard deviation or n (%). Student’s t-test was used for comparisons of means, and the chi-square test was used to compare categorical variables between the two groups, as appropriate. Pearson’s correlation test was used for the correlation analyses. The receiver operating characteristics (ROC) curve of cathepsin B in predicting severe preeclampsia was analyzed. The results were considered significant if p values were <0.05, and highly significant if p<0.01.

## Results

One hundred forty pregnant women were enrolled in the study, of which 100 subjects were preeclamptic (late-onset preeclampsia diagnosed at ≥34 weeks’ gestation) and 40 were healthy controls. Twenty-six subjects in the preeclamptic group had severe features.

Cathepsin B concentrations were significantly higher in the preeclamptic group compared with the control group (4.24±3.51 ng/mL vs. 2.04±1.97 ng/mL, respectively; p<0.001). Cathepsin D concentrations were significantly higher in the preeclamptic group compared with the control group (4.97±1.24 ng/mL vs. 4.20±1.65 ng/mL, respectively; p<0.01). There was no statistically significant difference between the groups in terms of cathepsin L concentrations (Table 1). 

There were no statistically significant differences between the groups in terms of age, body mass index, and gestational age. Systolic and diastolic blood pressures and serum uric acid concentrations were significantly higher in the preeclamptic group (Table 1). The mean value for 24-hour urine protein in the preeclamptic group was 2980.12±2516.33 mg/24 h. Cathepsin B concentrations were found to be positively correlated with uric acid concentrations (r=0.343, p<0.01) in women with preeclampsia. Cathepsin B concentrations were significantly higher in women with preeclampsia with severe features (n=26) compared with those with preeclampsia alone (6.89±3.51 ng/mL vs. 3.31±3.02 ng/mL, respectively; p<0.001). The ROC curve of cathepsin B in predicting severe preeclampsia was analyzed. The area under the curve of cathepsin B was 0.81 (95% CI: 0.71-0.90) and the optimal cut-off level was 4.71 ng/mL, yielding 85% sensitivity and 74% specificity. 

## Discussion

Preeclampsia is a multifactorial clinical state that adversely affects several vital organs and increases the morbidity and mortality of both the fetus and the mother^(7,8,9)^. Although there is growing evidence and there are many theories addressing its heterogeneous nature, the pathogenesis is not yet fully understood^(7,8,9)^.

The aim of the study was to evaluate cathepsin concentrations in women with late-onset preeclampsia and to determine the impact of cathepsins with regard to the presence of severe features. To our knowledge, this is the first study to emphasize the importance of cathepsin B, D, and L serum concentrations in women with late-onset preeclampsia.

In our study, we found significantly higher serum concentrations of cathepsin B and D in the preeclamptic group compared with the control group; however, cathepsin L concentrations were similar between the two groups. Moreover, cathepsin B concentrations were found to be positively correlated with uric acid concentrations in women with preeclampsia. Our data also indicate that cathepsin B concentrations were significantly higher in women with preeclampsia with severe features compared with those with preeclampsia alone, emphasizing the importance of cathepsin B in women with preeclampsia and in the subgroup of preeclamptic women with severe features.

Cysteine cathepsins are lysosomal peptidases that comprise cathepsin B and cathepsin L, which have many physiologic roles in different organs and tissues including cancer progression, tumor proliferation triggers, invasion, and metastasis,^(10,11)^ and it has been demonstrated that abnormal concentrations and activities may correlate with various physiologic processes and human diseases, such as neurodegenerative disorders,^(12)^ regulation of apoptosis, immune responses, inflammatory diseases,^(13)^ cancer,^(11,14,15)^ psoriasis,^(16)^ and cardiovascular^(17,18)^ and kidney diseases^(19)^.

In a study evaluating serum concentrations of cathepsin B and L that included 40 women with preeclampsia and 38 women as controls, higher concentrations of cathepsin B and L were reported in women with preeclampsia; however, no statistically significant difference was reported between women with severe preeclampsia compared with subjects with mild preeclampsia^(20)^. The distribution and abnormal expression concentrations of cysteine cathepsins were also reported in preeclamptic placentas indicating their important roles during normal placentation and in the etiology of preeclampsia^(1)^.

Circulating concentrations of cathepsin B and D were determined in a cohort of 72 pregnant women in which 25 were preeclamptic and 47 were normotensive. In accordance with our study, cathepsin B concentrations were found to be significantly increased in preeclamptic women, whereas in contrast to our data, no significant difference was found in cathepsin D concentrations between the preeclamptic and control groups, and no correlation was found between the cathepsin concentrations and the severity of preeclampsia^(21)^. The differential expression or aberrant release of cathepsin proteases from trophoblasts or other types of cells were suggested to have a key role in the pathophysiology of preeclampsia^(21)^.

Cathepsin D is a lysosomal aspartic proteinase that plays a key role in protein degradation and in apoptotic processes that are induced by oxidative stress, cytokines, and aging^(22)^. Few studies have evaluated the impact of cathepsin D in women with preeclampsia. In a study evaluating cathepsin D activity in the umbilical cord, it was reported that preeclampsia was associated with a reduction in the activity of cathepsin D in human umbilical cord^(5)^. The placentas of preeclamptic subjects were also evaluated by Kim et al.,^(23)^ and an overexpression of cathepsin D in the placentas from preeclamptic patients was demonstrated suggesting a trigger of apoptosis. In another study, circulating serum concentrations of cathepsin D were found to be significantly lower in the preeclamptic group (n=15) than in normotensive pregnancies (n=35) and also similar to those in non-pregnant healthy patients (n=20); however, a limited number of preeclamptic cases were included in the study and further studies with larger sample sizes were suggested^(4)^. In our study, we found that serum cathepsin D concentrations were significantly higher in the preeclamptic group compared with the control group.

### Study Limitations

The limitation of the study is its small sample size. Further studies with larger sample sizes are needed.

## Conclusion

This is the first study to demonstrate the impact of cathepsin B, D, and L on late-onset preeclampsia. There are few previously published studies with small sample sizes that have reported conflicting results about the importance of cathepsins in the pathogenesis of preeclampsia. The present study shows that women with late-onset preeclampsia have significantly higher serum cathepsin B and D concentrations than controls, and cathepsin B concentrations are even higher in the subgroup of preeclampsia that has severe features. In summary, the study suggests that cathepsin B and D may be promising biomarkers in women with late-onset preeclampsia. Moreover, cathepsin B may be useful in early identification of preeclamptic women with severe features.

## Figures and Tables

**Table 1:**
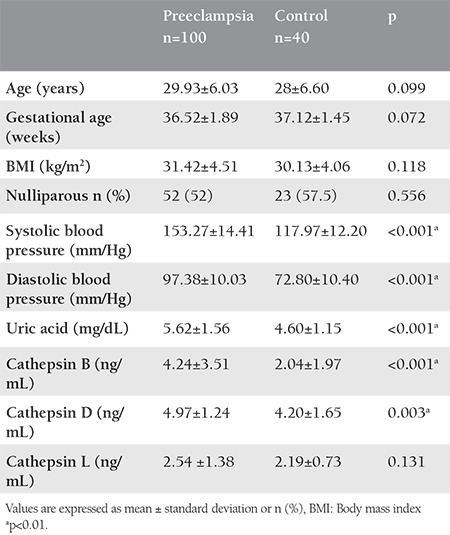
Clinical and biochemical characteristics of the groups

## References

[1] Varanou A, Withington SL, Lakasing L, Williamson C, Burton GJ, Hemberger M (2006). The importance of cysteine cathepsin proteases for placental development. J Mol Med (Berl).

[2] Lisonkova S, Sabr Y, Mayer C, Young C, Skoll A, Joseph KS (2014). Maternal morbidity associated with early-onset and late-onset preeclampsia. Obstet Gynecol.

[3] Amarante-Paffaro AM, Hoshida MS, Yokota S, Gonçalves CR, Joazeiro PP, Bevilacqua E, et al (2011). Localization of cathepsins D and B at the maternal-fetal interface and the invasiveness of the trophoblast during the postimplantation period in the mouse. Cells Tissues Organs.

[4] Kim HY, Lee M, Kang HW, Moon C (2013). Cathepsin D concentrations are reduced in patients with preeclampsia in Korean population. Clin Biochem.

[5] Galewska Z, Bańkowski E, Romanowicz L, Gogiel T, Wolańska M, Jaworski S (2005). Preeclampsia-associated reduction of cathepsin D activity in the umbilical cord. Clin Chim Acta.

[6] American College of Obstetricians and Gynecologists; Task Force on hypertension in pregnancy (2013). Hypertension in pregnancy. Report of the American College of Obstetricians and Gynecologists’ Task Force on hypertension in pregnancy. Obstet Gynecol.

[7] Armaly Z, Jadaon JE, Jabbour A, Abassi ZA (2018). Preeclampsia: Novel mechanisms and potential therapeutic approaches. Front Physiol.

[8] Lisowska M, Pietrucha T, Sakowicz A (2018). Preeclampsia and related cardiovascular risk: Common genetic background. Curr Hypertens Rep.

[9] Jim B, Karumanchi SA (2017). Preeclampsia: Pathogenesis, prevention, and long-term complications. Semin Nephrol.

[10] Anja P, Anahid J, Janko K (2018). Cysteine cathepsins: their biological and molecular significance in cancer stem cells. Semin Cancer Biol.

[11] Mohamed MM, Sloane BF (2006). Cysteine cathepsins: multifunctional enzymes in cancer. Nat Rev Cancer.

[12] Pišlar A, Kos J (2014). Cysteine cathepsins in neurological disorders. Mol Neurobiol.

[13] Conus S, Simon HU (2008). Cathepsins: key modulators of cell death and inflammatory responses. Biochem Pharmacol.

[14] Olson OC, Joyce JA (2015). Cysteine cathepsin proteases: regulators of cancer progression and therapeutic response. Nat Rev Cancer.

[15] Jedeszko C, Sloane BF (2004). Cysteine cathepsins in human cancer. Biol Chem.

[16] Kawada A, Hara K, Kominami E, Hiruma M, Noguchi H, Ishibashi A (1997). Processing of cathepsins L, B and D in psoriatic epidermis. Arch Dermatol Res.

[17] Liu CL, Guo J, Zhang X, Sukhova GK, Libby P, Shi GP (2018). Cysteine protease cathepsins in cardiovascular disease: from basic research to clinical trials. Nat Rev Cardiol.

[18] Li X, Liu Z, Cheng Z, Cheng X (2012). Cysteinyl cathepsins: multifunctional enzymes in cardiovascular disease. Chonnam Med J.

[19] Cocchiaro P, De Pasquale V, Della Morte R, Tafuri S, Avallone L, Pizard A, et al (2017). The multifaceted role of the lysosomal protease cathepsins in kidney disease. Front Cell Dev Biol.

[20] Dong M, Wang H, Huang H (2007). Alterations of serum cathepsins B and L in pre-eclampsia. Clin Chim Acta.

[21] Kim HY, Kim BW, Kim YJ (2016). Elevated serum cathepsin B concentration in pregnant women is associated with preeclampsia. Arch Gynecol Obstet.

[22] Haendeler J, Popp R, Goy C, Tischler V, Zeiher AM, Dimmeler S (2005). Cathepsin D and H2O2 stimulate degradation of thioredoxin-1: implication for endothelial cell apoptosis. J Biol Chem.

[23] Kim YN, Kim HK, Warda M, Kim N, Park WS, Prince Adel B, et al (2007). Toward a better understanding of preeclampsia: comparative proteomic analysis of preeclamptic placentas. Proteomics Clin Appl.

